# Sex and age differences in chronic postoperative pain among patients undergoing thoracic surgery: a retrospective cohort study

**DOI:** 10.3389/fmed.2023.1180845

**Published:** 2023-06-07

**Authors:** Ying Zhao, Xin-Min Liu, Lu-Yao Zhang, Bing Li, Ruo-Han Wang, Qin-Yue Yuan, Shi-Chao Wang, Hai-Peng Zhu, Hui Zhi, Jia-Qiang Zhang, Wei Zhang

**Affiliations:** ^1^Department of Anesthesiology and Perioperative Medicine, Zhengzhou University People’s Hospital; Henan Provincial People’s Hospital, Zhengzhou, Henan, China; ^2^Department of Anesthesiology and Perioperative Medicine, Henan University People’s Hospital; Henan Provincial People’s Hospital, Zhengzhou, Henan, China

**Keywords:** chronic postoperative pain, sex, thoracic surgery, retrospective cohort, dose response relationship

## Abstract

**Background:**

The effect of sex and age on chronic post-thoracic surgical pain (CPTP) at rest and with activity remains unclear. The main purpose of this study was to investigate the relationship between the incidence of chronic postoperative pain (at rest and with activity) and sex/age differences.

**Methods:**

This was a single-center retrospective study that included adult patients who had undergone elective thoracic surgery. Patients were divided into two groups based on sex. Demographic and perioperative data were collected, including age, sex, education level, Body Mass Index (BMI), American Society of Anesthesiologists (ASA) physical status, and medical history (hypertension, diabetes mellitus). Chronic postoperative pain data were collected by telephone follow-up.

**Results:**

Among the 3,159 patients enrolled, 1,762 were male, and 1,397 were female. After creating a matched-pairs cohort, 1,856 patients were analyzed. The incidence of CPTP at rest was 14.9% among males and 17.8% among females (*p* = 0.090). The incidence of CPTP with activity was 28.4% among males and 35.0% among females (*p* = 0.002). We analyzed three different models after propensity matching to validate the stability of the prediction model between sex and CPTP, and female sex was a significant predictor of CPTP with activity 3 months after surgery. Further analysis showed that females in the 45-55-year-old age group were more prone to develop CPTP.

**Conclusion:**

Females have a higher incidence of chronic postoperative pain with activity after thoracic surgery. Females in the 45-55-year-old age group are more prone to develop CPTP than females in other age groups.

## Introduction

Chronic postsurgical pain (CPSP) is defined as the presence of surgery-related pain for more than 3 months postoperatively, not including pain due to explicit reasons (such as chronic infection and the recurrence of malignant tumors) (1, 2). The incidence of CPSP is reported to be 3–85%, which varies greatly for different types of surgery (3). The incidence of CPSP varies, and patients undergoing thoracic surgery have a higher rate of CPSP than patients undergoing other types of surgery (4). A meta-analysis of prospective studies reported that the incidence of chronic post-thoracic surgical pain (CPTP) at 3 months was 57% (1). CPTP affects patients’ daily lives and reduces their quality of life (5, 6).

Many studies have explored the relationship between sex and postoperative pain. According to previous studies, female sex was associated with chronic postoperative pain after thoracic surgery (7, 8). However, Tighe’s study showed that when examining each type of surgery, the effect of sex on postoperative pain was negligible (9). Therefore, the effect of sex on CPTP after thoracic surgery needs further study for verification. CPTP is usually divided into pain at rest and pain with activity. Pain with activity is defined as pain when coughing. To date, it is not clear how sex affect CPTP at rest and with activity.

In this study, we attempted to qualify whether sex is associated with the incidence of chronic postoperative pain after thoracic surgery, with the goal of identifying patients at risk, which might be employed to improve the experience of thoracic surgery patients. Through a large-sample retrospective study, we examined the impact of sex and explored the effect of age on the incidence of CPTP.

## Materials and methods

This single-center retrospective cohort study was conducted at Henan Provincial People’s Hospital using data from database and questionnaire-based sources. The study involving human participants were reviewed and approved by the Ethical Committee of Henan Provincial People’s Hospital (Approval No.100). Data on patient characteristics were collected from the Information Center Department of Henan Provincial People’s Hospital.

We adopted the definition of CPTP formulated by the International Association for the Study of Pain (IASP) (2). At the 3 month follow-up, the diagnosis of CPTP was made by asking patients about the presence of pain localized to their surgical incision that was not present before surgery or caused by any other known cause, such as infection or malignancy. Patients were asked to report the intensity of pain at rest and with activity (when coughing) via a telephone review. CPTP was defined when the pain intensity, measured by an 11-point numeric rating scale (NRS), was higher than 1 point at 3 months after surgery (10). The pain intensity was classified as no pain (NRS = 0), mild pain (NRS = 1–3), moderate pain (NRS = 4–6), and severe pain (NRS = 7–10). In addition, we used the Quality of Recovery 15 (QoR-15) score to evaluate the quality of recovery via telephone follow-up (11). The score assesses five domains of patient-reported health status (pain, physical comfort, physical independence, psychological state, and emotional state) to fully assess a patient’s recovery experience. At 3 months after thoracic surgery, all eligible patients were interviewed *via* telephone. The telephone follow-up was conducted by a well-trained researcher who was blinded to our study to collect information on CPTP and the quality of recovery.

### Study population

Patients aged 18 years or older who were admitted for elective thoracic surgery between January 2018 and June 2020 were enrolled in this study. Patients were excluded for the following reasons: (1) mental illness or communication disorders; (2) loss to follow-up for 3 months after surgery; (3) death within the first 3 months after surgery; (4) undergoing a second surgery within the first 3 months after surgery; (5) metastasis and relapse of cancer; and (6) inadequate acute pain scores from medical records.

### Anesthesia management and postoperative analgesic protocol

The protocol of anesthesia was in accordance with standard process of thoracic anesthesia in Henan provincial People’s Hospital. Anesthesia and perioperative protocol were identical between the two groups. All the patients received intravenous anesthesia combined with inhalation anesthesia. For perioperative analgesia, multimodal analgesia management was widely used. Multiple nerve block methods (including thoracic paravertebral block, serratus anterior plane block, etc.) are used for intraoperative analgesia. At the end of surgery, patient controlled intravenous analgesia (PCIA) would be applied for postoperative analgesia.

### Variables

Variables chosen for CPTP were selected based on clinical experience and literature reviews (7, 12). Preoperative variables included the following patient demographics: age, sex, education level, Body Mass Index (BMI), American Society of Anesthesiologists (ASA) physical status, tobacco exposure history, drinking history, and medical history (hypertension, diabetes mellitus). Intraoperative variables included the use of regional anesthesia, intraoperative sentinel consumption, surgical characteristics (surgical method, surgery type and surgery duration), the amount of bleeding, and urine volume. Postoperative variables included LOS in the PACU, LOS in the hospital, the use of PCIA, the use of postoperative salvage analgesics, and postoperative pain at rest and with activity at the first 24 postoperative hour.

### Endpoint and confounders

The primary purpose of the current study was to investigate differences in the incidence of CPTP in males and females who underwent thoracic surgery. The secondary endpoint of the study was to investigate the differences in the quality of recovery at 3 months postoperatively between males and females and the differences in the effect of age on the incidence of CPTP in females and males.

Baseline factors thought to have relationships with CPTP were regarded as potential confounders for the analysis. Based on clinical experience and previous studies, we adjusted for the potential confounding effects of age, ASA physical status, surgery type, surgical incision, the use of regional anesthesia, the use of PCIA, postoperative pain at the first 24 h, postoperative complications, tobacco exposure history and intraoperative sufentanil consumption. All information concerning potential confounders was retrieved from electronic medical records.

### Statistical analysis

All statistical analyses were performed using the Statistical Package for the Social Sciences (SPSS, version 26.0, IBM Corporation, NY) and R (version 4.2.0). Baseline data were divided into two groups based on sex. Continuous variables are presented as the mean ± standard deviation (if the data are normal) or as the median ± interquartile range, and categorical variables are expressed as absolute values and percentages. We conducted analyses on baseline patient demographics between males and females using a t test for continuous variables and a chi-square test for categorical variables.

Since this was a retrospective database study, the number of eligible patients was fixed. Therefore, we estimated the statistical power rather than calculating the sample size. We used propensity score matching (PSM) to exclude systematic bias. Patients were matched using 1:1 nearest-neighbor matching with a caliper size of 0.05 on a propensity score scale. Patients were matched on the following covariates: age, BMI, ASA physical status, education level, tobacco exposure history, drinking history, history of hypertension, surgery duration, surgery type, surgery method, the dosage of intraoperative sufentanil, the use of PCIA, and the use of regional anesthesia. The covariate balance was estimated by calculating the standardized mean differences (SMDs). A good balance was considered for a SMD <0.1. An SMD value of 0.1–0.3 was considered to indicate a small difference; a value above 0.3 was considered to indicate a moderate to considerable difference. To control the influence of covariates with *p* value less than 0.05 after matching, we incorporated these variables into the final multivariable logistic regression model to analyze the association between the exposure and outcome. To test the robustness of our main findings, we conducted an *a priori*-defined sensitivity analysis. As stated above, 3 analysis models were devised: “Model 1” was a crude model; “Model 2” was adjusted for age, sex, ASA physical status and tobacco exposure history; and “Model 3” was adjusted for age, ASA physical status, surgery type, surgical method, the use of regional anesthesia, the use of PCIA, tobacco exposure history, acute pain at rest and with activity during the first 24 postoperative hours, postoperative complications and intraoperative sufentanil consumption.

The associations of age with the incidence of CPTP with activity were analyzed with restricted cubic spline curves for females and males. A dose–response relationship model was used to analyze the effect of age on CPTP with activity, and 95% confidence intervals (CIs) were calculated.

## Results

### Characteristics of patients

**Figure 1:**
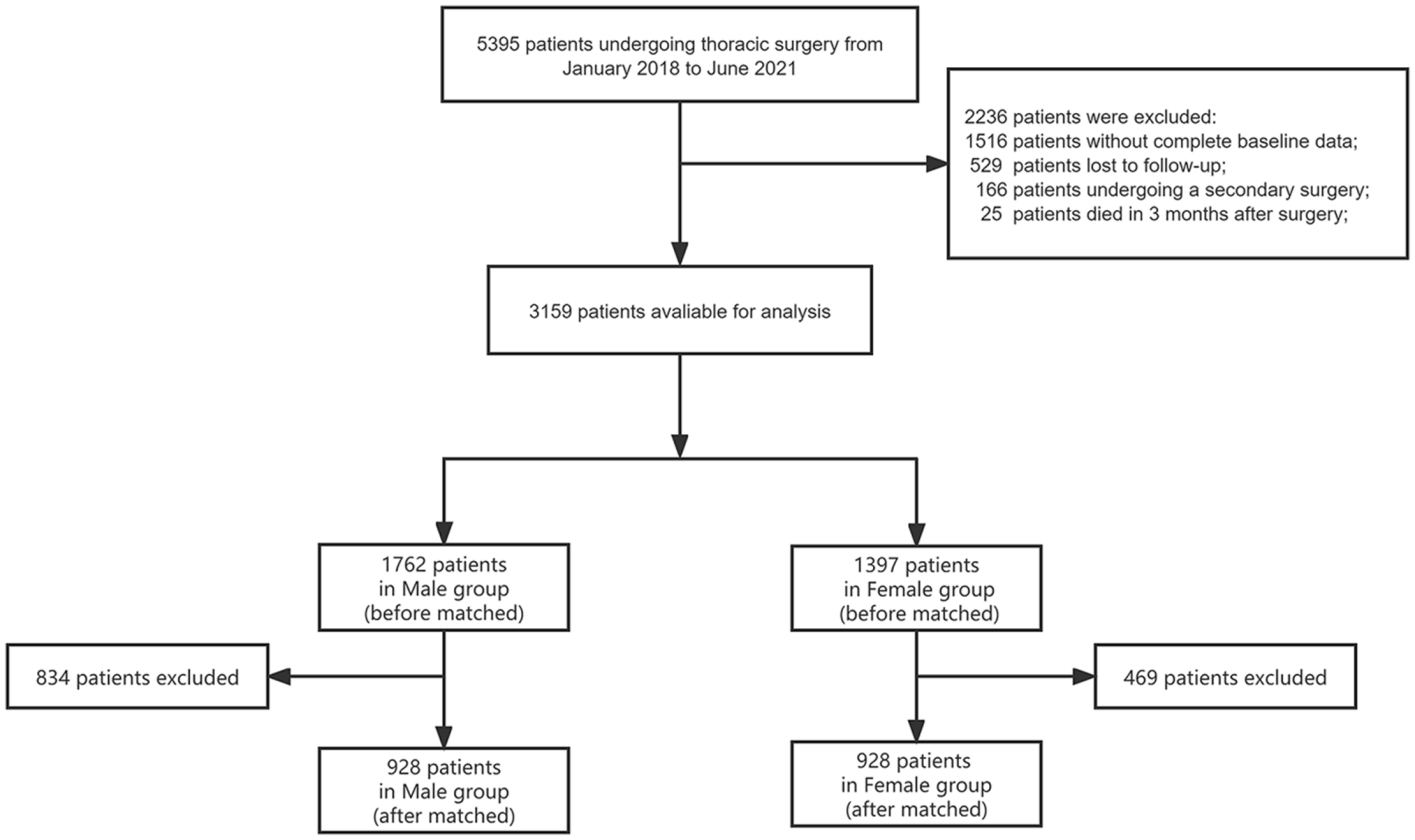
Flow chart of this study.

Of the 5,395 patients who underwent elective thoracic surgery identified in our database, 3,159 (58.6%) were eligible for inclusion. A total of 1,238 (22.9%) patients were excluded due to missing baseline data, and 168 (3.1%) patients were excluded for undergoing a second surgery during the follow-up period. At the 3-month follow-up period, 794 (14.7%) patients were unable to be contacted, and 36 (0.7%) patients had died. Ultimately, a total of 3,159 patients were enrolled for final analysis (Figure 1).

The demographic characteristics are summarized in Table 1. The two groups were comparable in tobacco exposure history (*p* = 0.031) after the propensity-score matched-pairs analysis. The intraoperative data are summarized in Table 2. Notably, the consumption of intraoperative sufentanil was higher in male group (40.0[30.0, 40.0]) than female group (35.0[30.0, 40.0], *p* = 0.002) and there was significant difference between the groups in surgery type (*p* = 0.002) after the propensity-score matched-pairs analysis.

**Table 1:** Patient characteristics.

Items	Before matched	After matched	*p*	Male *n* = 928	Female *n* = 928	*p*	SMD
	Male *n* = 1762	Female *n* = 1,397					
BMI (kg/㎡)	21.7 (20.0, 24.0)	21.6 (19.4, 24.7)	0.756	21.9 (20.2, 24.1)	21.7 (19.4, 24.9)	0.272	0.051
Age (years old)	57.0 (49.0, 65.0)	56.0 (48.0, 65.0)	0.017	57.0 (50.0, 65.0)	56.0 (49.0, 65.0)	0.742	0.015
ASA physical status			<0.001			0.584	0.067
I	36 (2.0)	35 (2.5)		22 (2.4)	16 (1.7)		
II	1,385 (78.6)	1,162 (83.2)		745 (80.3)	756 (81.5)		
III	340 (19.3)	200 (14.3)		160 (17.2)	156 (16.8)		
IV	1 (0.05)	0 (0)		1 (0.1)	0 (0)		
Education			<0.001			0.132	0.165
Illiteracy	61 (3.5)	80 (5.8)		37 (4.0)	57 (6.1)		
Primary school	584 (33.8)	514 (37.4)		330 (35.6)	342 (36.9)		
Middle school	727 (42.1)	518 (37.8)		393 (42.3)	364 (39.2)		
Bachelor or above	356 (20.6)	261 (19.0)		168 (18.1)	165 (17.8)		
Tobacco exposure history(yes)	848 (48.1)	474 (34.0)	<0.001	415 (44.8)	368 (39.7)	0.031	0.103
Drinking history (yes)	750 (42.7)	31 (2.2)	<0.001	21 (2.3)	21 (2.3)	1	<0.001
History of hypertension (yes)	256 (14.2)	185 (13.2)	0.261	96 (10.3)	111 (12.0)	0.268	0.055
Diabetes history (yes)	100 (5.6)	68 (4.9)	0.315	44 (4.7)	46 (5.0)	0.914	0.010

Data are presented as the mean (SD) or n (%) or median (IQR).

SD, standard deviation; IQR, interquartile range; BMI: body mass index; ASA, American Society of Anesthesiologists.

**Table 2:** Baseline data of intraoperative patients.

Items	Before matched			After matched			SMD
	Male *n* = 1762	Female *n* = 1,397	*p*	Male *n* = 928	Female *n* = 928	*p*	
Intraoperative sufentanil (μg)	40.0 (30.0, 40.0)	32.5 (30.0, 40.0)	<0.001	40.0 (30.0, 40.0)	35.0 (30.0,40.0)	0.002	0.108
Surgery time (min)	210.0 (164.0, 270.0)	190.0 (155.0, 240.0)	<0.001	210.0 (160.0, 266.5)	200.0 (160.0, 255.0)	0.071	0.084
Amount of bleeding (mL)	100.0 (50.0, 100.0)	50.0 (50.0, 100.0)	<0.001	100.0 (50.0, 100.0)	100.0 (50.0, 100.0)	0.293	0.049
Urine volume (mL)	400.0 (200.0, 600.0)	300.0 (200.0, 600.0)	0.209	400.0 (200.0, 600.0)	400.0 (200.0, 600.0)	0.079	0.082
Regional anesthesia (yes)	1,512 (85.8)	1,135 (81.2)	<0.001	783 (84.3)	771 (83.1)	0.489	0.035
Type of regional anesthesia			0.202			0.453	
TPVB	1,441 (81.8)	1,087 (77.8)		746 (80.4)	737 (79.4)		
ESPB	16 (0.9)	12 (0.9)		11 (1.2)	11 (1.2)		
SAPB	5 (0.3)	10 (0.7)		4 (0.4)	9 (0.9)		
TAP	35 (2.0)	17 (1.2)		14 (1.5)	9 (0.9)		
ICNB	15 (0.9)	9 (0.6)		8 (0.8)	5 (0.5)		
PCIA (yes)	1,615 (91.6)	1,275 (91.3)	0.696	820 (88.4)	837 (90.2)	0.230	0.059
Surgical method (Endoscopic surgery)	1,552 (88.0)	1,324 (94.8)	<0.001	843 (90.8)	858 (92.5)	0.240	0.058
Surgery type			<0.001			0.002	0.182
Lung cancer and Lobectomy	1,146 (65.0)	1,048 (75.0)		625 (67.3)	670 (72.2)		
Esophageal cancer surgery	343 (19.5)	138 (9.9)		169 (18.2)	115 (12.4)		
Mediastinal surgery	142 (8.1)	146 (10.5)		77 (8.3)	97 (10.5)		
Other types	131 (7.4)	65 (4.7)		57 (6.1)	46 (5.0)		

Data are presented as the mean (SD) or *n* (%) or median (IQR).

TPVB, thoracic paravertebral block; SAPB, Serratus anterior plane block; TAP, Transversus abdominis plane block; ICNB, Intercostal nerve block; SD, standard deviation; IQR, interquartile range; PCIA, patient controlled intravenous analgesia.

### Primary outcome

**Figure 2:**
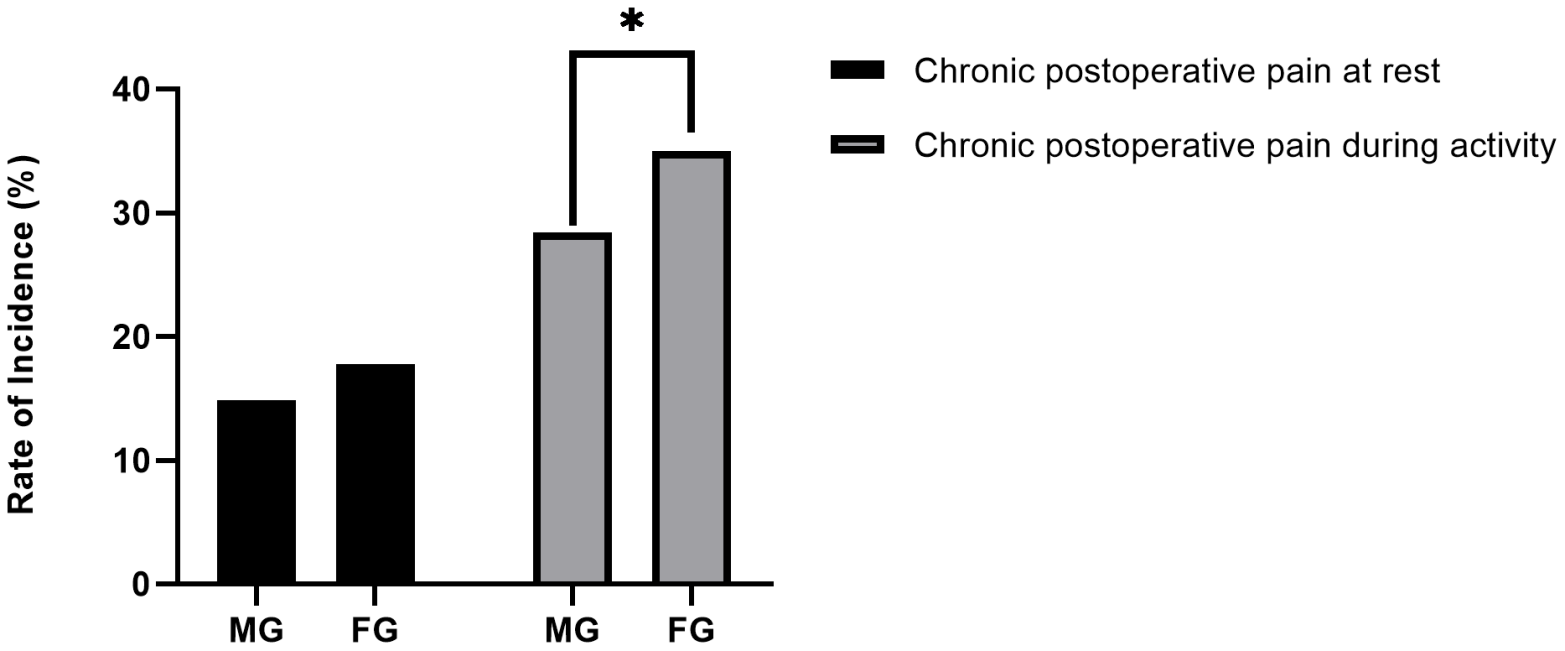
Incidence of CPTP in males and females. The ordinate is the incidence rate. Categorical variables were analyzed by the chi-square test. There was a statistically significant difference in chronic pain with activity between the two groups. *p < 0.05. FG, Female Group; MG, Male Group; CPTP, Chronic Post-thoracotomy Pain.

The incidence of CPTP with activity in female (35.0%) was higher than that in male (28.4%), indicating that more patients in female group who underwent CPTP with activity (*p* = 0.043, Figure 2). However, there was no difference in the incidence of CPTP at rest between the two groups (17.8% vs. 14.9%, *p* = 0.090) (Figure 2). Furthermore, the NRS score of CPTP at rest (0.0 [0.0–0.0] vs. 0.0 [0.0–0.0], *p* = 0.043) and that with activity (0.0 [0.0–1.0] vs. 0.0 [0.0–1.0], *p* = 0.000) were higher in female group than those in male group.

### Secondary outcome

There were no significant differences between male and female group in terms of incidence of postoperative acute pain at rest (49.6% vs. 48.3%, *p* = 0.577) or that with activity (71.8% vs. 72.3%, *p* = 0.976, Table 3) in the first postoperative 24 h. Similarly, there was no difference in the score of Qor-150 [150 (140, 150) vs. 148 (140, 150), *p* = 0.334] between the two groups (Table 3). However, there were differences between the two groups in length of stay in the post-anesthesia care unit [65.0(49.3, 85.0) vs. 67.5(50.0, 95.0), *p* = 0.017], length of stay in the hospital [8.0 (6.0, 10.0) vs. 7.0 (6.0, 10.0), *p* < 0.001] and postoperative complications (66.1% vs. 59.1%, *p* = 0.002, Table 3).

**Table 3:** Patient data after surgery.

Items	Before matched			After matched		
	Male *n* = 1762	Female *n* = 1,397	*p*	Male *n* = 928	Female *n* = 928	*p*
LOS in PACU (min)	70.0 (55.0, 90.0)	70.0 (55.0, 95.0)	0.009	65.0 (49.3, 85.0)	67.5 (50.0, 95.0)	0.017
LOS in hospital (day)	8.0 (6.0, 10.0)	7.0 (6.0, 9.0)	<0.001	8.0 (6.0, 10.0)	7.0 (6.0, 10.0)	<0.001
Postoperative salvage analgesics (yes)	19 (1.1)	22 (1.6)	0.221	9 (1.0)	15 (1.6)	0.218
Postoperative chemotherapy (yes)	540 (30.7)	416 (29.8)	0.598	292 (31.4)	277 (29.8)	0.450
Postoperative radiotherapy (yes)	13 (0.7)	5 (0.4)	0.162	8 (0.9)	4 (0.4)	0.247
Postoperative complications (yes)	1,139 (66.1)	797 (58.8)	<0.001	613 (66.1)	548 (59.1)	0.002
Acute pain at rest						
Incidence (%)	843 (47.8)	668 (47.8)	0.988	460 (49.6)	448 (48.3)	0.577
NRS (score)	0.0 (0.0, 2.0)	0.0 (0.0, 2.0)	0.899	0.0 (0.0, 2.0)	0.0 (0.0, 2.0)	0.585
The severity of acute pain at rest			0.175			0.290
None	919 (52.2)	729 (52.2)		468 (50.4)	480 (51.1)	
Light	719 (40.8)	576 (41.2)		383 (41.3)	391 (41.7)	
Moderate	93 (5.3)	80 (5.7)		61 (6.6)	48 (5.9)	
Severe	31 (1.8)	12 (0.9)		16 (1.7)	9 (1.3)	
Acute pain with activity						
Incidence (%)	1,269 (72.0)	1,015 (72.7)	0.692	666 (71.8)	671 (72.3)	0.796
NRS (score)	2.0 (0.0, 3.0)	2.0 (0.0, 3.0)	0.756	2.0 (0.0, 3.0)	2.0 (0.0, 3.0)	0.946
The severity of acute pain with activity			0.450			0.541
None	493 (28.0)	382 (27.3)		262 (28.2)	257 (27.7)	
Light	987 (56.0)	814 (58.2)		523 (56.4)	542 (58.4)	
Moderate	228 (12.9)	168 (12.1)		114 (12.3)	109 (11.1)	
Severe	54 (3.1)	33 (2.4)		29 (3.1)	20 (2.2)	
CPTP at rest						
Incidence (%)	246 (13.7)	250 (17.9)	0.003	138 (14.9)	165 (17.8)	0.090
NRS (score)	0.0 (0.0, 0.0)	0.0 (0.0, 0.0)	0.001	0.0 (0.0, 0.0)	0.0 (0.0, 0.0)	0.043
The severity of CPTP at rest			0.011			0.170
None	1,516 (86.0)	1,147 (82.2)		790 (85.1)	763 (82.2)	
Light	227 (12.9)	228 (16.3)		129 (13.9)	150 (16.2)	
Moderate	19 (1.1)	21 (1.5)		9(1.0)	15 (1.6)	
Severe	0 (0)	0 (0)		0(0)	0 (0)	
CPTP with activity						
Incidence (%)	478 (27.1)	469 (33.6)	<0.001	264(28.4)	325 (35.0)	0.002
NRS (score)	0.0 (0.0, 1.0)	0.0 (0.0, 1.0)	0.000	0.0(0.0, 1.0)	0.0 (0.0, 1.0)	0.000
The severity of CPTP with activity			<0.001			0.001
None	1,283 (72.8)	928 (66.4)		664 (71.6)	603 (65.0)	
Light	453 (25.7)	426 (30.5)		252 (27.2)	293 (31.6)	
Moderate	24 (1.4)	39 (2.8)		10 (1.1)	30 (3.2)	
Severe	2 (0.1)	4 (0.3)		2 (0.2)	2 (0.2)	
QoR-15 (score)	150.0 (140.0, 150.0)	148.0 (140.0, 150.0)	0.059	150.0 (140.0, 150.0)	148.0 (140.0, 150.0)	0.334

Data are presented as the mean (SD) or *n* (%) or median (IQR).

SD, standard deviation; IQR, interquartile range; LOS, length of stay; PACU, post anesthesia care unit; CPTP, chronic post-thoracotomy pain; QoR, quality of recovery.

### Models of the multivariable logistic regression analysis

**Figure 3:**
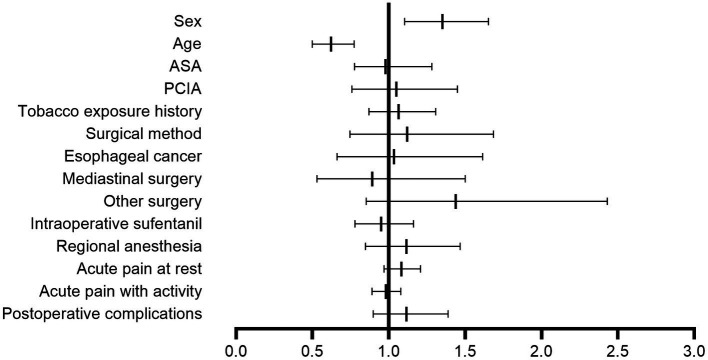
Forest plot of the multivariable logistic regression analysis for chronic postoperative pain with activity. The forest plot was drawn from the data obtained by the multivariable logistic regression equation, and older age (adjusted OR = 0.622, 95% CI, 0.500–0.733; *p* < 0.001) was a protective factor for chronic postoperative pain with activity. Females (adjusted OR = 1.351, 95% CI, 1.104–1.652; *p* = 0.003) were more likely to experience chronic postoperative pain than males. OR, odds ratio; CI, confidence interval; PCIA, patient-controlled intravenous analgesia; ASA, American Society of Anesthesiologists.

We analyzed three different models after PSM, including Model 1 (crude model, odds ratio [OR] = 1.356, 95% confidence interval [95% CI], 1.114 to 1.650; *p* = 0.002), Model 2 (adjusted for age, ASA physical status and tobacco exposure, adjusted odds ratio [adjusted OR] = 1.335, 95% CI, 1.095 to 1.627; *p* = 0.004), and Model 3 (adjusted for age, ASA physical status, tobacco exposure, surgery type, surgical method, intraoperative sufentanil, PCIA, regional anesthesia, acute pain at rest, acute pain with activity and postoperative complications; adjusted odds ratio [adjusted OR] = 1.351, 95% CI, 1.104 to 1.652; *p* = 0.003), to validate the stability of the prediction model between females and CPTP with activity (Table 4). By using the data from the regression equation, we plotted the forest plot (Figure 3).

**Table 4:** Multivariable logistic regression analysis for CPTP with activity.

Variable	B coefficient	Adjusted OR (95% CI)	*p*
Model 1	0.304	1.356 (1.114 to 1.650)	0.002
Model 2	0.289	1.335 (1.095 to 1.627)	0.004
Model 3	0.301	1.351 (1.104 to 1.652)	0.003

Model 1 was a crude model.

Model 2 was adjusted for age, ASA physical status and tobacco exposure.

Model 3 was adjusted for age, ASA physical status, tobacco exposure, surgery type, surgical method, intraoperative sufentanil, PCIA, regional anesthesia, acute pain at rest, acute pain with activity and postoperative complications. Abbreviations: OR, odds ratio; CI, confidence interval; ASA, American Society of Anesthesiologists; PCIA, patient controlled intravenous analgesia; CPTP, chronic post-thoracotomy pain; B coefficient, regression coefficient.

### Effect of age on CPTP in females and males

**Figure 4:**
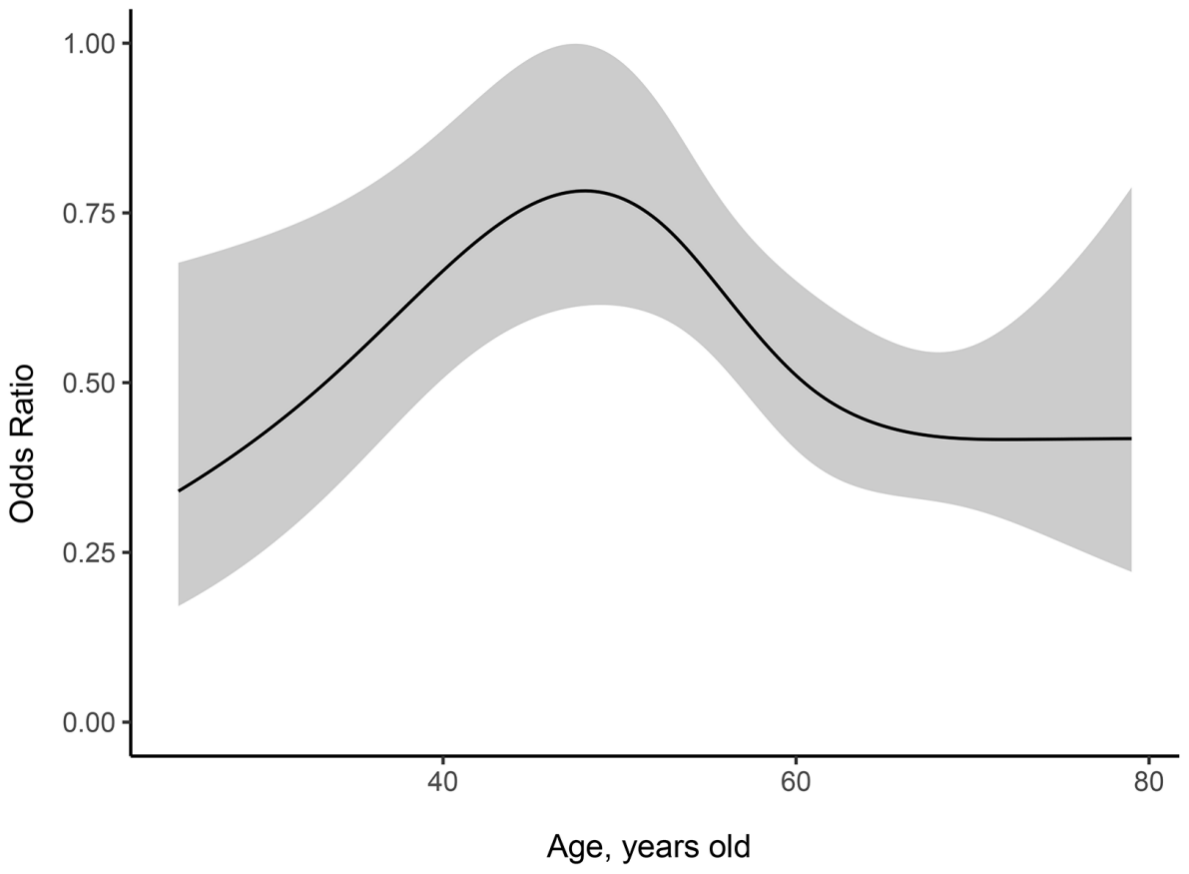
Dose–response relationship between age and CPTP with activity in females. The ordinate is the odds ratio (0–1), and the abscissa is female age (18–90 years old). Error bars represent 95% confidence intervals. The risk of CPTP with activity increased rapidly until the age range of 45 to 55 years and then decreased slowly. Females in the 45-55-year-old age group had the highest incidence of CPTP with activity compared with those in the other age groups. CPTP, Chronic Post-thoracotomy Pain.

**Figure 5:**
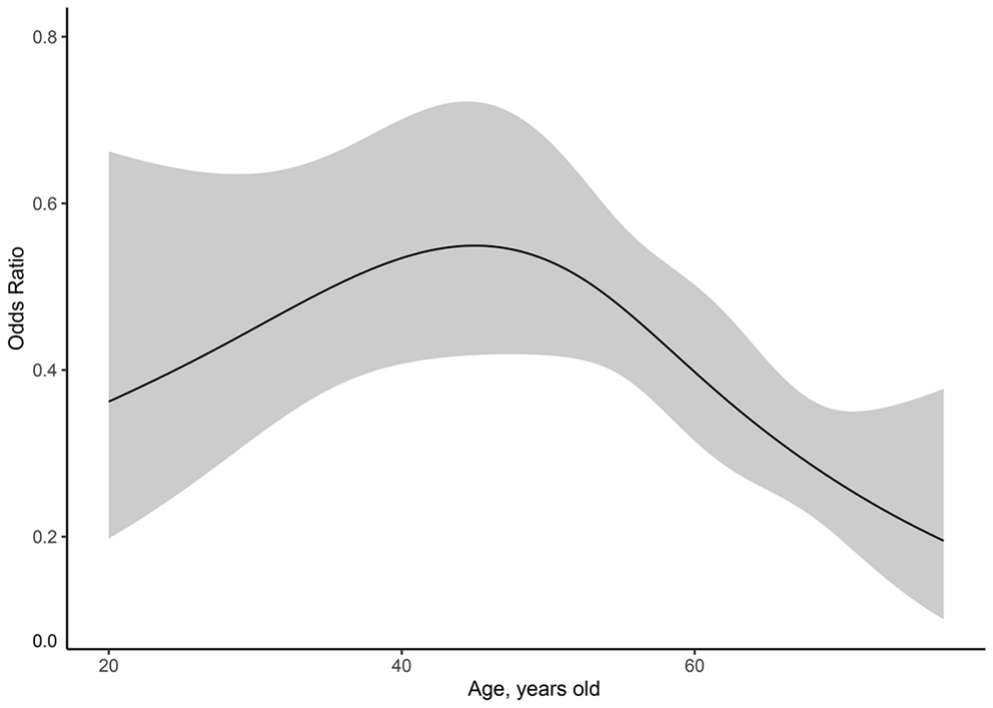
Dose–response relationship between age and CPTP with activity in males. The ordinate is the odds ratio (0–1), and the abscissa is the age of the male (18–90 years old). Error bars represent 95% confidence intervals. The risk of CPTP with activity increased with age from 18 years old, peaked at 30-55-year-old, and then decreased slowly in males. CPTP, Chronic Post-thoracotomy Pain.

We used restricted cubic splines to flexibly model and visualize the dose–response relationship between age and CPTP with activity in females (Figure 4). The ordinate was the OR (0–1), and the abscissa was female age (18–90 years old). The risk of CPTP with activity in females increased with age beginning at 18 years old, peaked at 45 to 55 years old, and then decreased slowly. A similar method was used to validate the effect of age on CPTP with activity in males (Figure 5). The risk of CPTP with activity in males increased slowly with age beginning at 18 years old, peaked at 35 to 55 years old, and then decreased slowly.

We then analyzed the effect of four different age ranges on CPTP with activity in females, including the 18-45-year-old (OR = 0.685, 95% CI, 0.454–1.027; *p* = 0.069), 55-60-year-old (OR = 0.603, 95% CI, 0.393–0.915; *p* = 0.019) and 60-90-year-old age groups (OR = 0.515, 95% CI, 0.325 to 0.726; *p* < 0.001) (Table 5). Compared with the other age groups, the incidence of CPTP with activity was the highest in females in the 45-55-year-old age group.

**Table 5:** Dose–response relationship model of different ages on CPTP with activity.

Group(sex)	Age(year)	B coefficient	OR (95% CI)	*p*
Female	18–45	−0.378	0.685(0.454 to 1.027)	0.069
	45–55		Reference	Reference
	55–60	−0.506	0.603(0.393 to 0.915)	0.019
	60–90	−0.664	0.515(0.325 to 0.726)	<0.001
Male	18–30	−0.772	0.462(0.235 to 0.857)	0.018
	30–55		Reference	Reference
	55–90	−0.594	0.552(0.402 to 0.758)	<0.001

B coefficient, regression coefficient; OR, odds ratio; CI, confidence interval; CPTP, chronic post-thoracotomy pain.

Finally, the effect of three different age groups on CPTP with activity was analyzed in males, including the 18-30-year-old (OR = 0.462, 95% CI, 0.235–0.857; *p* = 0.018) and 55-90-year-old age groups (OR = 0.552, 95% CI, 0.402–0.758; *p* < 0.001) (Table 5). Compared with the other age groups, the incidence of CPTP with activity was the highest in males in the 30-55-year-old age group.

## Discussion

In this study, we investigated the effect of sex on the incidence of CPTP and explored the age differences in CPTP. First, our study showed that among females, the incidence of CPTP with activity was 35.0%. Females experienced a higher incidence of CPTP with activity than males. Second, the incidence of CPTP with activity during perimenopause (between 45 and 55 years old) was 43.7%, which was the highest among all female age groups. Third, the incidence of CPTP in adult women increased beginning at 18 years old, peaked at 45–55 years old, and then gradually declined.

Through a large-sample retrospective study, we reviewed the data of patients receiving who underwent thoracic surgery over a period of 4 years at Henan Provincial People’s Hospital, including esophageal surgery, lung surgery, mediastinal surgery and other thoracic surgeries. We further determined the relationship between sex and CPTP. Moreover, we analyzed three different models after PSM and showed that female sex was a risk factor for CPTP with activity, further supporting that this prediction model was very stable, which is consistent with previous research.

As a highly prevalent complication following thoracic surgery, chronic postsurgical pain has been investigated for decades. Previous systematic and nonsystematic reviews have shown that female sex is a risk factor for chronic postoperative pain after thoracotomy (7, 13). However, CPTP has not been discussed from two different perspectives (pain at rest and pain with activity), and most previous studies had small sample sizes. In addition, we divided CPTP into CPTP at rest and CPTP with activity to study the effect of sex on CPTP, which was one of the advantages of our study. Our study showed that when chronic postoperative pain was divided into pain at rest and pain with activity, only CPTP with activity showed differences between females and males.

Our study demonstrated that females tended to experience a higher incidence of CPTP with activity than males. There are some reasonable interpretations for this result. First, men and women have completely different levels of sex hormones. Paige’s study demonstrated that the duration of mechanical hypersensitivity was dependent on circulating sex hormones in mice, where estrogen caused an extension of sensitivity and testosterone was responsible for a decrease in duration in the chronic pain model (14). Second, some studies (15, 16) have shown that anxiety and depression may be related to some clinical pain, and women are more prone to these negative emotions than men, which may also be a factor leading to sex differences in pain (17).

It is worth mentioning that females in the 45-55-year-old group were more prone to develop CPTP than females in other age groups in the current study. This age group is highly coincident with perimenopause (18). Martin’s review showed that most chronic noncancer pain patients displayed significant increases in pain prevalence in the reproductive years between puberty and menopause, which suggests that ovarian hormones may be responsible for the differences in the prevalence (19). Perimenopause is characterized by dramatic fluctuations in hormone levels, especially in the levels of estrogen, which can persistently decrease, as expected, but can also abnormally increase (20, 21). Lbrahi’s study showed that hormonal instability during the perimenopausal period could not only cause vasomotor symptoms and mood disturbances but also lead to susceptibility to pain (22). An increasing trend of CPTP incidence from 18–45 years of age was found in our study. A similar finding was observed among males. Our study found that CPTP with activity first increased at 18 years old, peaked at 30–55 years old and then decreased after 55 years old in males. Kristensen reported that the prevalence of chronic pain among children and young adults after thoracotomy was lower if the surgery was performed at a younger age, which may be related to physiological and psychological factors (23). Similarly, another retrospective cohort study reported that the incidence rate of chronic postoperative pain in young individuals (aged ≤24 years old) was significantly lower than that in adults (aged >24 years old) who underwent the Nuss procedure for pectus excavatum (24). We further found that there was a gradual downward trend in the incidence of CPTP with increasing age after 55 years of age. This result was similar to Dijk’s study, which showed that pain scores decreased significantly with age in elderly people undergoing surgery (25).

An interesting finding of the current study is that there was a significant difference between females and males in the incidence of postoperative complications, LOS in the PACU and LOS in the hospital after surgery. According to a retrospective study, male sex was a significant risk factor for postoperative complications after lung cancer surgery (26), which was similar to the findings of the current study. Furthermore, the differences in postoperative complications between females and males may lead to differences in the length of hospital stay. Additionally, a significant difference in LOS in the PACU was observed between females and males. However, this difference was slightly small, and its clinical significance needs to be further explored.

We recognize that there were several limitations to this study. First, because of the retrospective design of the study, we could not obtain detailed information for some variables, such as preexisting pain conditions and psychological status, which are known risk factors for CPTP (27, 28). Second, the study’s follow-up period was limited to 3 months after surgery. However, a study conducted in Korea showed that 3.7% of patients were diagnosed with CPSP at least 6 months after surgery (29). Third, because of the limitation of follow-up by telephone, 14.7% of the patients were lost to follow-up. However, the number of patients excluded from our study was not higher than those who refused to participate or were excluded for other reasons in previous prospective studies (30).

Some studies have investigated the relationship between hormones changes and chronic pain in women, which showed that female patients responded well to hormone replacement therapy in relieving pain (31, 32). Our study demonstrated that female patients in 45-55-year-old were more susceptible to CPTP, which suggested that we should pay more attention to the incidence of CPTP in such a population. What’s more, we hypothesize that it may be a strategy to help female patients in perimenopause relieve chronic postoperative pain by hormone replacement therapy. However, the specific therapeutical program still needs more studies in the future.

In conclusion, female sex was a risk factor for CPTP with activity after elective thoracic surgery. The incidence of CPTP with activity increased with age beginning at 18 years old, peaked at 45–55 years old and then gradually decreased. Females in the 45-55-year-old age group had the highest incidence of CPTP with activity compared with those in the other age groups. The underlying mechanism needs further research.

## Data availability statement

The raw data supporting the conclusions of this article will be made available by the authors, without undue reservation.

## Ethics statement

The studies involving human participants were reviewed and approved by Ethics Committee of Henan Provincial People’s Hospital. Since this article is a retrospective study, there was no need to sign an informed consent.

## Author contributions

YZ wrote the manuscript. YZ and X-ML collected data. L-YZ performed bioinformatics analyses. S-CW and H-PZ analyzed the data. BL created the diagrams. HZ, R-HW, and Q-YY provided supervision. J-QZ and WZ made many contributions to the design of the research, performed data analyses, and graph generation, and wrote the manuscript. All authors contributed to the article and approved the submitted version.

## Funding

This study was supported by grants from the Medical Science and Technology Research Plan Joint Construction Project of Henan Province (LHGJ20200059), the Henan Province Middle-aged and Young Health Science and Technology Innovation Outstanding Youth Talent Training Project (YXKC2021025).

## Conflict of interest

The authors declare that the research was conducted in the absence of any commercial or financial relationships that could be construed as a potential conflict of interest.

## Publisher’s note

All claims expressed in this article are solely those of the authors and do not necessarily represent those of their affiliated organizations, or those of the publisher, the editors and the reviewers. Any product that may be evaluated in this article, or claim that may be made by its manufacturer, is not guaranteed or endorsed by the publisher.
